# Analyses of AUC_(0–12)_ and C_0_ Compliances within Therapeutic Ranges in Kidney Recipients Receiving Cyclosporine or Tacrolimus

**DOI:** 10.3390/jcm9123903

**Published:** 2020-12-01

**Authors:** Aurelija Radzevičienė, Pierre Marquet, Rima Maslauskienė, Rūta Vaičiūnienė, Edmundas Kaduševičius, Edgaras Stankevičius

**Affiliations:** 1 Institute of Physiology and Pharmacology, Medical Academy, Lithuanian University of Health Sciences, 9 A. Mickevičiaus Street, LT-44307 Kaunas, Lithuania; edmundas.kadusevicius@lsmuni.lt (E.K.); edgaras.stankevicius@lsmuni.lt (E.S.); 2 INSERM UMR 850, 87025 Limoges, France; pierre.marquet@unilim.fr; 3 Department of Pharmacology and Toxicology, CHU Limoges, 2, Avenue Martin Luther King, 87042 Limoges CEDEX, France; 4 Faculty of Medicine, University of Limoges, 87000 Limoges, France; 5 Department of Nephrology, Medical Academy, Lithuanian University of Health Sciences, 9 A. Mickevičiaus Street, LT-44307 Kaunas, Lithuania; rima.maslauskiene@lsmuni.lt (R.M.); ruta.vaiciuniene@lsmuni.lt (R.V.); 6 Institute of Cardiology, Lithuanian University of Health Sciences, 9 A. Mickevičiaus Street, LT-44307 Kaunas, Lithuania

**Keywords:** immunosuppression, AUC, C_0_, C_0_/AUC_(0–12)_ ratio, cyclosporine and tacrolimus

## Abstract

The AUC (area under the concentration time curve) is considered the pharmacokinetic exposure parameter best associated with clinical effects. Unfortunately, no prospective studies of clinical outcomes have been conducted in adult transplant recipients to investigate properly the potential benefits of AUC_(0–12)_ monitoring compared to the C_0_-guided therapy. The aim of the present study was to compare two methods, C_0_ (through level) and AUC_(0–12)_ (area under the concentration time curve), for assessing cyclosporine and tacrolimus concentrations. The study included 340 kidney recipients. The AUC_(0–12)_ was estimated using a Bayesian estimator and a three-point limited sampling strategy. Therapeutic drug monitoring of tacrolimus performed by using AUC_(0–12)_ and C_0_ showed that tacrolimus in most cases is overdosed when considering C_0_, while determination of the AUC_(0–12)_ showed that tacrolimus is effectively dosed for 27.8–40.0% of patients receiving only tacrolimus and for 25.0–31.9% of patients receiving tacrolimus with MMF (mycophenolate mofetil). In the 1–5 years post-transplantation group, 10% higher CsA (cyclosporine) dose was observed, which was proportionate with a 10% higher AUC_(0–12)_ exposure value. This indicates good compatibility of the dosage and the AUC(0–12) method. The Bland–Altman plot demonstrated that C_0_ and AUC_(0–12)_ might be interchangeable methods, while the ROC (receiver operating characteristic) curve analysis of the C_0_/AUC_(0–12)_ ratio in the tacrolimus-receiving patient group demonstrated reliable performance to predict IFTA (interstitial fibrosis and tubular atrophy) after kidney transplantation, with an ROC curve of 0.660 (95% confidence interval (CI): 0.576–0.736), *p* < 0.01. Moreover, AUC_(0–12)_ and C_0_ of tacrolimus depend on concomitant medication and adjustment of the therapeutic range for AUC_(0–12)_ might influence the results.

## 1. Introduction

Due to the narrow immunosuppressants’ therapeutic range, highly variable pharmacokinetics, inter-individual differences, and a tendency of interactions mainly occurring during the metabolism, which are cytochrome P450 3A4 (CYP3A4)-mediated therapeutic window and fairly common adverse effects [1,2], prescription of immunosuppressive medication requires continual follow-up of the drug blood concentration. Short-term outcomes are better tailored versus the long-term ones despite implementation of various strategies into clinical practice. Protocol biopsies, pharmacogenetics, and other assays have been developed to guide tailoring of immunosuppression; although promising results have been obtained, trials showing their ability to improve long-term outcomes are lacking and urgently needed [3]. Different follow-up techniques in various clinics make it more complicated. The standard care guidelines after kidney transplantation involve pharmacokinetic monitoring. It is not standardized and can be performed using diverse techniques, as C_0_, C_2_, and the area under the concentration time curve (AUC). There were significant differences in C_0_, C_2_, and the calculated AUC after shifting to single daily dosing of cyclosporine [4] The AUC might be calculated in accordance to the trapezoidal rule, the Bayesian or other model and gauge different time points [5], different clinical covariates [6], which leads to inter-clinical and inter-laboratory variability [7].

The AUC is considered the pharmacokinetic exposure parameter best associated with clinical effects. Unfortunately, no prospective studies of clinical outcomes have been conducted in adult and pediatric transplant recipients to investigate properly the potential benefits of AUC_(0–12)_ monitoring compared to the C_0_-guided therapy. In the present study, two pharmacokinetic methods, the C_0_ and the AUC_(0–12)_, for assessing cyclosporine and tacrolimus concentrations were analyzed, as well as these methods’ ability to contribute to determining the risk of kidney alteration in long-term treated patients.

## 2. Materials and Methods

### 2.1. Study Patients

**Figure 1:**
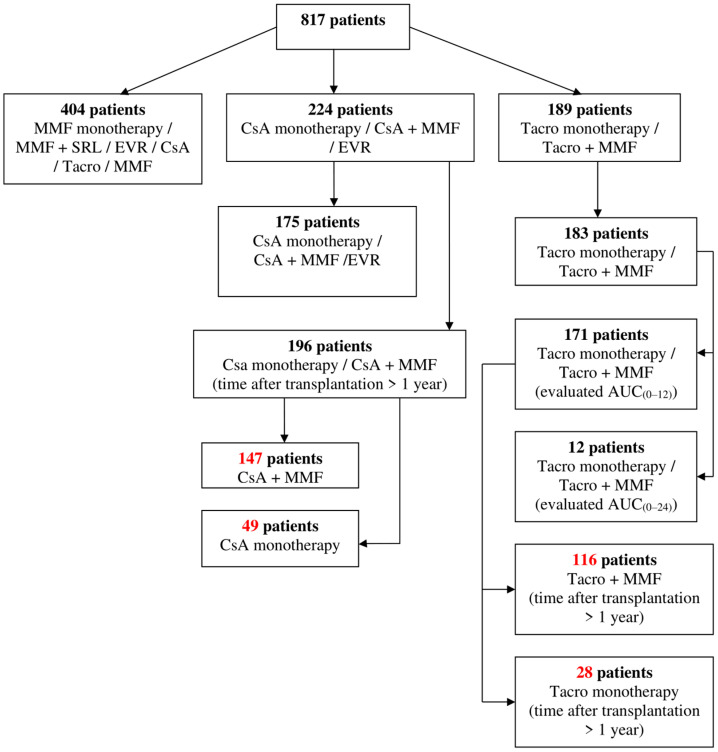
Patient screening. MMF, mycophenolate mofetil; SRL, sirolimus; EVR, everolimus; CsA, cyclosporine; Tacro, tacrolimus; AUC, area under the concentration time curve.

Anonymized medical records of 817 patients receiving immunosuppressant therapy after renal transplantation hospitalized at the Limoges University Hospital (France) during the study period from 2011 to 2012 were reviewed. Brief patient screening is available in Figure 1.

Renal transplant recipients aged from 19 to 83 years who underwent immunosuppressant (cyclosporine or tacrolimus) monitoring at the university hospital of Limoges during a 1-year period were included in the study.

The inclusion criteria were age of more than 18 years, stable kidney transplant, and immunosuppression with either cyclosporine or tacrolimus. Patients were excluded if they received immunosuppression with other medicaments and/or underwent transplantation of other organs. All patients received prednisolone orally according to the standard hospital practice. Protocol biopsies were taken to identify interstitial fibrosis and tubular atrophy (IFTA) [8,9].

The work described was carried out in accordance with the Code of Ethics of the World Medical Association (Declaration of Helsinki).

### 2.2. PK Sampling

For PK sampling, patients were admitted to hospital. After a first blood sampling for trough concentration, approximately 12 h after the evening dose, the fasting patients were given cyclosporine/tacrolimus and mycophenolate mofetil (if not monotherapy) doses at a precise time by the nurse. Then, blood samples were collected at 60 min ± 15 min and 180 min ± 30 min after administration into EDTA-containing vacutainers.

### 2.3. Determination of CsA

The blood samples were collected in EDTA (ethylenediaminetetraacetic acid) tubes to measure the CsA trough level (C_0_) and drug blood concentration 1 (C_1_) and 3 (C_3_) hours after the use of a CsA dose. CsA whole blood concentrations were measured using a validated turbulent flow chromatography–tandem mass spectrometry technique [10]. Online extraction was performed at 1.25 mL/min^−1^ using Cyclone P^®^, a 50 μm particle size (50 × 0.5 mm, i.d.) column (Thermo Fisher, Waltham, MA, USA), in alkaline conditions. Chromatographic separation was performed in acidic conditions (phase A: 0.1% formic acid in water; phase B: 0.1% formic acid in methanol) using Propel MS C18, a 5 μm (50 × 3.0 mm, i.d.) column (Thermo Fisher) kept at 60 °C with a constant flow rate of 300 μL/min^−1^. Detection was performed using a TSQ Quantum Discovery tandem mass spectrometer equipped with an orthogonal electrospray ionization source and controlled by the XCalibur software (Thermo Fisher). Tandem mass spectrometry detection was performed in the positive ion multiple reaction monitoring (MRM) mode following 3 transitions for cyclosporine (m/z 1220.0→1203.0 for quantification and m/z 1220.0→1185.0 and m/z 1220.0→425.0 for confirmation) and 2 transitions (m/z 1234.0→1217.0 for quantification and m/z 1234.0→119.0 for confirmation) for its analog cyclosporine D used as an internal standard (IS). Methanol/aqueous zinc sulphate (200 μL, 70:30 *v*/*v*) containing the internal standard at 25 μg/L^−1^ was added to the whole blood (100 μL). The mixture was vortex-mixed for 45 s, centrifuged at 13,000 rpm, and the supernatant was introduced into a 200 μL vial for injection. Calibration standards at 0, 10, 20, 50, 100, 200, 500, 1000, and 2000 μg/L^−1^ of CsA were prepared by spiking blank blood. The limits of detection (LOD) and quantification (LOQ) were 10 μg/L^−1^ and 20/μg L^−1^, and the calibration curves obtained using quadratic regression from the LOQ to 2000 μg/L^−1^ yielded r^2^ > 0.99.

### 2.4. Determination of Tacrolimus

Tacrolimus was determined using a previously reported validated turbulent flow chromatography–tandem mass spectrometry (TFC–MS/MS) method [11]. Briefly, online extraction was performed at 1.25 mL/min^−1^ using Cyclone P^®^, a 50 mm particle size (50 × 0.5 mm, i.d.) column (Cohesive Technologies, Milton Keynes, UK) in alkaline conditions. Chromatographic separation was performed in acidic conditions using Propel MS C18, a 5 mm (50 × 3.0 mm, i.d.) column (Cohesive Technologies, Milton Keynes, UK) heated to 60 °C, with a constant flow rate of 300 mL/min^−1^. Detection was performed using a TSQ Quantum Discovery MS/MS system (Thermo Fisher, Les Ulis, France) equipped with an orthogonal electrospray ionization source and controlled by the XCalibur software (Thermo Fisher). MS/MS detection was performed in the positive ion, multiple reaction monitoring mode following two transitions for tacrolimus (m/z 821.5→768.6; m/z 821.5→786.4) and two for the internal standard ascomycin (m/z 809.3→756.4; m/z 809.3→564.4).

This method was fully validated for tacrolimus determination in whole blood. The calibration curves using a 1/x weighted quadratic regression were used to obtain the best fit across the calibration range, based on the standard error of the fit and minimization of the calibrator’s bias. The lower limit of quantification was 1 mg/L^−1^ and the calibration curves obtained from the lower limit of quantification up to 100 mg/L^−1^ yielded r^2^ > 0.998. The method was found to be accurate and precise with the bias from −4.4% to 0.6% and a low coefficient of variation from −3.8% to 6.4% [11].

### 2.5. Pharmacokinetic Analysis

The NONMEM^®^ version VI (GloboMax^®^ LLC, Budapest, Hungary) nonlinear mixed-effects population pharmacokinetic model and the Bayesian estimator of a three-point limited sampling strategy developed at the Limoges University Hospital were used to determine cyclosporine [12,13] and tacrolimus [14] area under the blood concentration/time curve (AUC_(0–12)_). Briefly, concentration data obtained in de novo or stable renal transplant patients were fitted using a one-compartment open model with first-order elimination combined with a gamma model of absorption with two parallel absorption routes; or a two-compartment open model with first-order elimination and an Erlang absorption model. Based on these PK models, several maximum a posteriori Bayesian estimators allowing the determination of individual PK parameters and AUC estimation were developed using the iterative two-stage Bayesian approach and/or nonlinear mixed-effects modeling. For each of them, either external or internal validation (i.e., in independent groups of patients or using data splitting or the bootstrap approach) was performed before any clinical use.

### 2.6. Statistical Analysis

The G∗Power 3.1.9.4 version was used to calculate the sample size. Statistical test, MANOVA (multivariate analysis of variance), with effect size of f2(V) = 0.07 was used. The total calculated sample size was 136 patients with an actual power of 0.95 for each group: cyclosporine-receiving patients and tacrolimus-receiving patients.

Statistical analysis was performed using IBM SPSS 20.0. Pharmacokinetic parameters (AUC_(0–12)_ and C_0_) of CsA and Tacro were assessed (compliance within the therapeutic ranges) and compared between the patients’ groups. The unpaired *t*-test was used to compare the study groups (GraphPad software, available online: http://www.graphpad.com/quickcalcs/ttest1.cfm). Probability values of less than 0.05 were considered significant. GraphPad was used for scatter plots and MedCalc—for ROC analyses. ROC analyses were performed looking for cut-off values.

The Bland–Altman approach is used for assessing equivalence in method comparison studies. Bland–Altman plots were calculated using IBM SPSS 20.0 following the methods published by Davide Giavarina [15] and Nurettin Özgür Doğan [16]. Two methods are considered interchangeable if their differences are not clinically significant. The second approach is based on the errors-in-variables regression in a classical (X,Y) plot and focuses on confidence intervals, whereby two methods are considered equivalent when providing similar measures notwithstanding the random measurement errors. Bland–Altman plots were used to demonstrate how frequently the C_0_ assessment is insufficient for the calcineurin inhibitor monitoring based on the AUC.

### 2.7. Definition of Non-Compliance

Tacrolimus or cyclosporine AUC_(0–12)_ exposure or C_0_ values outside the determined therapeutic range were evaluated as non-compliance in this study. Therapeutic range of the AUC_(0–12)_ exposure was 120–150 µg/h/L for tacrolimus and 3.05–3.75 mg/h/L and 2.70–2.98 mg h/L for cyclosporine post-transplantation, respectively. The therapeutic range of C_0_ was 4–6 µg/L and 3–5 µg/L for tacrolimus 1–5 years and >5 years post-transplantation, respectively; and 75–150 µg/L for cyclosporine. Therapeutic ranges of AUC_(0–12)_ and C_0_ are summarized in Table 1.

## 3. Results

In total, 340 kidney recipients were enrolled: 196 kidney recipients received cyclosporine (CsA) and 144 kidney recipients received tacrolimus (Tacro), post-transplantation time > 1 year, 2 BID (twice (two times) a day) regimens.

### 3.1. Baseline Data of Study Patients Receiving Tacrolimus

Tacrolimus-receiving patients’ group consisted of 144 patients: 116 patients were tacrolimus- and MMF-treated, and 28 patients were tacrolimus-treated only. Patient age varied from 19 to 75 years, mean: 53.31 ± 12.31 (SD) years. All patients were stable kidney recipients with post-transplantation time > 1 year (1.00–20.30 years, mean: 5.16 ± 3.78 (SD) years). The Tacro dose varied from 1.0 to 13.0 mg/day, mean: 4.99 ± 2.40 (SD) mg/day. These patients were divided into four following groups depending on the post-transplantation time (1–5 years after transplantation and >5 years after transplantation) and the medication they received (Tacro + MMF or Tacro monotherapy). For the detailed information, see Table 2.

AUC_(0–12)_ exposure and C_0_ values were assessed in these groups. The gauge was compliance within the therapeutic range. The summarized data of the AUC_(0–12)_ exposure compliance within the therapeutic range in the patients receiving tacrolimus is provided in Table 3. The summarized data of the C_0_ compliance within the therapeutic range in the patients receiving tacrolimus is provided in Table 4. In most cases, the evaluation of Tacro C_0_ demonstrated that Tacro C_0_ values were not in compliance within the established therapeutic ranges and Tacro was prescribed in high doses or overdosed. Compliance within the therapeutic range rates (60.0%) was only observed in the patients on tacrolimus monotherapy (post-transplantation time: 1–5 years).

To note, there is only one therapeutic range for the AUC_(0–12)_ assessment (120–150 µg h/L) in all groups of patients receiving Tacro, while there are several therapeutic ranges for the assessment of C_0_. Therapeutic ranges of C_0_ depend on the post-transplantation time. The initial treatment doses were higher and the therapeutic width was in a higher range for early post-transplant stage patients. The dose of the immunosuppressive drug was reduced and the therapeutic width was adjusted within switching to maintenance treatment. Therapeutic ranges for both AUC_(0–12)_ and C_0_ assessments are given in Table 2.

### 3.2. Baseline Data of Study Patients Receiving Cyclosporine

The cyclosporine-treated patients’ group consisted of 196 subjects: 147 patients were cyclosporine- and MMF-treated, and 49 patients were cyclosporine-treated only. Patient age varied from 22 to 83 years, mean: 58.26 ± 13.85 (SD) years. All patients were stable kidney recipients with post-transplantation time > 1 year (1.00–26.24 years, mean: 9.63 ± 6.12 (SD) years). The CsA dose varied from 60 to 400 mg/day, mean: 185.00 ± 52.53 (SD) mg/day. These patients were divided into four following groups depending on the post-transplantation time (1–5 years after transplantation and >5 years after transplantation) and the medication they received (CsA + MMF or CsA monotherapy). The baseline data of each cyclosporine-treated group are provided in Table 5.

The evaluation included cyclosporine dosing, compliance and non-compliance rates of AUC_(0–12)_ and C_0_ within the therapeutic ranges of AUC_(0–12)_ and/or C_0_. To assess CsA concentrations in blood and the calculated AUC_(0–12)_, the previously determined therapeutic latitudes for the AUC_(0–12)_ exposure were used [17] instead of the AUC_(0–12)_ targeting to 3.8 mg/L.

### 3.3. Comparison of C_0_ and AUC_(0–12)_ Methods for Assessing Tacrolimus Concentrations in Tacrolimus-Receiving Subjects with Post-Transplantation Time from 1 to 5 Years and with Post-Transplantation Time > 5 Years

#### 3.3.1. Analysis of the AUC_(0–12)_ Compliance within the Therapeutic Range

The evaluation of AUC_(0–12)_ demonstrated higher compliance within therapeutic range rates in the patients receiving only tacrolimus (27.8–40.0%) versus the patients receiving tacrolimus together with MMF, where compliance rates were 25.0–31.9%. Low dosing was more often observed in the patients receiving only tacrolimus (44.4–60.0%), while higher rates of overdosing were observed in the patients receiving tacrolimus with MMF (29.5–36.1%) (Table 3).

Low tacrolimus dosing is often associated with transplanted organ rejection. This study’s data showed that around 45% of the patients with grafts older than 5 years had AUC_(0–12)_ exposure below the range, which was possibly related to low tacrolimus dosing in the maintenance state and higher transplanted organ rejection risk. This risk remained increased regardless of whether patients received Tacro monotherapy or Tacro with MMF (44.4% vs. 45.5%). The patients receiving only Tacro with grafts aged 1–5 years were at an even higher organ rejection risk (60.0%). The results are presented in Table 3.

#### 3.3.2. Analysis of the C_0_ Compliance within the Therapeutic Range

Slightly different results were obtained while analyzing the C_0_ compliance within the therapeutic range. The most accurate dosing was observed in the patients with grafts aged 1–5 years receiving Tacro monotherapy (60.0%). Cases of tacrolimus overdosing or cases when tacrolimus C_0_ values were above the therapeutic range were observed in the patients with grafts aged 1–5 years receiving Tacro with MMF (81.9%); in patients with grafts aged > 5 years receiving Tacro + MMF or only Tacro—93.2% and 88.9%, respectively. The results are presented in Table 4.

The data showed that the C_0_ assessment presented more cases of Tacro overdosing than of accurate dosing, while the AUC_(0–12)_ assessment presented more cases of Tacro overdosing or low dosing. The results are presented in Table 3 and Table 4.

#### 3.3.3. Independent Sample *t*-Test Analyses

**Figure 2:**
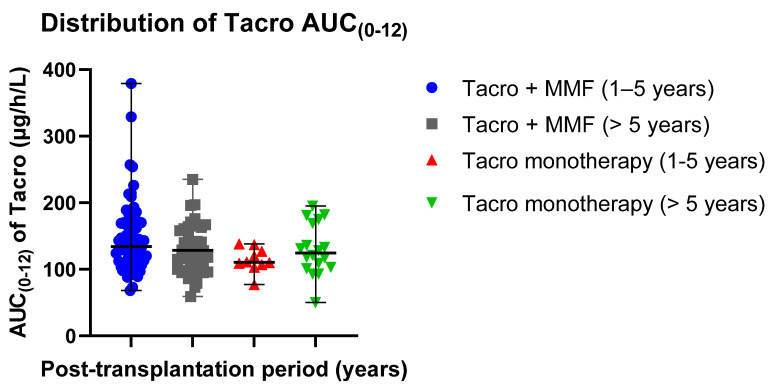
Distribution of the Tacro AUC _(0–12)_ exposure values in the study groups: Tacro + MMF and Tacro monotherapy; MMF—mycophenolate mofetil; Tacro—tacrolimus. Scatter plots display the individual AUC_(0–12)_ exposure values’ distribution from the median in the Tacro-receiving study groups (median ± SE): 134.00 ± 6.251 µg/h/L (Tacro + MMF, post-transplantation time: 1–5 years) versus 110.50 ± 5.675 µg/h/L (Tacro monotherapy, post-transplantation time: 1–5 years); 128.50 ± 5.532 µg/h/L (Tacro + MMF, post-transplantation time > 5 years) versus 124.50 ± 8.978 µg/h/L (Tacro monotherapy, post-transplantation time > 5 years). Respectively, mean values (mean ± SD) were as follows: 146.97 ± 53.039 µg/h/L (Tacro + MMF, post-transplantation time 1–5 years) versus 113.90 ± 17.947 µg/h/L (Tacro monotherapy, post-transplantation time: 1–5 years); 129.73 ± 36.695 µg/h/L (Tacro + MMF, post-transplantation time > 5 years) versus 129.72 ± 38.092 µg/h/L (Tacro monotherapy, post-transplantation time > 5 years).

The distribution of mean AUC_(0–12)_ exposure values in different groups was 146.97 ± 53.04 µg/h/L in patients receiving Tacro with MMF (post-transplantation time: 1–5 years) and 113.90 ± 17.95 µg/h/L in patients receiving Tacro monotherapy (post-transplantation time: 1–5 years); 129.73 ± 36.70 µg/h/L in patients receiving Tacro with MMF (post-transplantation time > 5 years) and 129.72 ± 38.09 µg h/L in patients receiving Tacro monotherapy (post-transplantation time > 5 years) (Table 2). Data distribution is presented in scatter plots (Figure 2) displaying individual AUC _(0–12)_ values’ distribution from the median in Tacro-receiving study groups (median ± SE): 134.00 ± 6.251 µg/h/L (Tacro + MMF, post-transplantation time: 1–5 years) versus 110.50 ± 5.675 µg/h/L (Tacro monotherapy, post-transplantation time: 1–5 years); 128.50 ± 5.532 µg/h/L (Tacro + MMF, post-transplantation time > 5 years) versus 124.50 ± 8.978 µg/h/L (Tacro monotherapy, post-transplantation time > 5 years). An independent sample *t*-test showed that the data for the groups receiving tacrolimus with MMF or only tacrolimus statistically significantly differ in patients with the post-transplantation time of 1–5 years. These results suggested that Tacro AUC_(0–12)_ statistically significantly depends on the concomitant medication used with Tacro, either MMF or no MMF (*p* = 0.047; *p* < 0.05).

**Figure 3:**
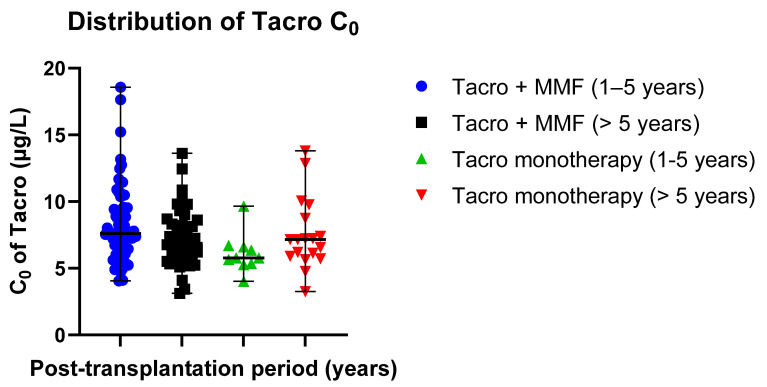
Distribution of Tacro C_0_ values in the study groups: Tacro + MMF and Tacro monotherapy; MMF—mycophenolate mofetil; Tacro—tacrolimus. Scatter plots display the individual C_0_ values’ distribution from the median in the Tacro-receiving study groups (median ± SE): 7.62 ± 0.33 µg/L (Tacro + MMF, post-transplantation time: 1–5 years) versus 5.77 ± 0.46 µg/L (Tacro monotherapy, post-transplantation time: 1–5 years); 6.88 ± 0.32 µg/L (Tacro + MMF, post-transplantation time > 5 years) versus 7.16 ± 0.63 µg/L (Tacro monotherapy, post-transplantation time > 5 years). Respectively, mean values (mean ± SD): 8.23 ± 2.81 µg/L (Tacro + MMF, post-transplantation time: 1–5 years) versus 6.12 ± 1.47 µg/L (Tacro monotherapy, post-transplantation time: 1–5 years); 7.23 ± 2.14 µg/L (Tacro + MMF, post-transplantation time > 5 years) versus 7.54 ± 2.67 µg h/L (Tacro monotherapy, post-transplantation time > 5 years).

Mean Tacro C_0_ values were as follows: 8.23 ± 2.81 µg/L (post-transplantation time: 1–5 years, patients receiving Tacro + MMF) versus 6.12 ± 1.47 µg/L (post-transplantation time: 1–5 years, Tacro monotherapy); 7.23 ± 2.14 µg/L (post-transplantation time > 5 years, patients receiving Tacro + MMF) versus 7.54 ± 2.67 µg/L (post-transplantation time > 5 years, Tacro monotherapy) (Table 2). Data distribution is presented in scatter plots (Figure 3) displaying the individual C_0_ values’ distribution from the median in the Tacro-receiving study groups (median ± SE): 7.62 ± 0.33 µg/L (Tacro + MMF, post-transplantation time: 1–5 years) versus 5.77 ± 0.46 µg/L (Tacro monotherapy, post-transplantation time: 1–5 years); 6.88 ± 0.32 µg/L (Tacro + MMF, post-transplantation time > 5 years) versus 7.16 ± 0.63 µg/L (Tacro monotherapy, post-transplantation time > 5 years).

Independent sample *t*-test results of C_0_ were compared in two different post-transplantation time groups: post-transplantation time of 1–5 years and post-transplantation time > 5 years. Patients were receiving either Tacro with MMF or Tacro monotherapy. Results of the independent sample *t*-test showed a statistically significant difference between mean C_0_ values in the post-transplantation time of 1–5 years groups, *p* = 0.023 (*p* < 0.5). These results confirm that the C_0_ value might depend on the concomitant medication used with Tacro, while no statistically significant difference between mean C_0_ values in the post-transplantation time > 5 years groups was not noticed, *p* = 0.635 (*p* > 0.5).

#### 3.3.4. ANOVA Analysis

ANOVA analysis of four tacrolimus-receiving patients’ groups showed that only C_0_ statistically significantly differed between the groups, *p* = 0.022 (*p* < 0.05) (Table 2).

#### 3.3.5. Rates of Non-Compliance

Patients using high medication doses (i.e., overdosing, non-compliance) are at higher risk of nephropathy, especially when the medication is nephrotoxic, like tacrolimus. Non-compliance was associated with the increased risk of interstitial fibrosis and tubular atrophy. The results are presented in Table 6 and Table 7.

The assessment of C_0_ enabled to identify a larger number of subjects with interstitial fibrosis and tubular atrophy (IFTA) than the assessment of AUC_(0–12)_. IFTA was obtained in 17 out of 72 (23.6%) patients receiving Tacro + MMF (post-transplantation time: 1–5 years). Non-compliance was obtained in 13 out of 17 (76.5%) patients with C_0_ > 6 µg/L. The assessment of AUC_(0–12)_ enabled to identify 4 out of 17 patients (23.5%) with AUC_(0–12)_ > 150 µg/h/L. Similar results were obtained in other tacrolimus-receiving patients’ groups (Table 6 and Table 7).

#### 3.3.6. Cut-Offs for Non-Compliance

IFTA was observed in 34 (23.6%) patients out of 144 patients. ROC curve analyses were performed to identify the cut-off values of increased risk of Tacro-induced IFTA.

A cut-off of 95.00 µg/h/L was the optimal one to maximize AUC_(0–12)_ sensitivity and specificity for IFTA. According to this cut-off, 18 (12.50%) patients’ AUC_(0–12)_ values were below this threshold, and in 11 (61.10%) of these cases, IFTA was observed, while among the 126 patients with the AUC_(0–12)_ value > 95.00 µg/h/L, 23 (18.30%) corresponded to IFTA. This cut-off corresponds to a sensitivity of 32.35% and a specificity of 93.64%.

A cut-off of 6.21 µg/L was the optimal one to maximize C_0_ sensitivity and specificity for IFTA. According to this cut-off, 43 (29.86%) patients’ C_0_ values were below this threshold, and in 14 (32.60%) of these cases, IFTA was observed, while among the 101 patients with the C_0_ value > 6.21 µg/L, 20 (19.80%) corresponded to IFTA. This cut-off corresponds to a sensitivity of 41.18% and a specificity of 73.64%.

A cut-off of 0.059 was the optimal one to maximize the C_0_/AUC_(0–12)_ ratio’s sensitivity and specificity for IFTA. According to this cut-off, 84 (58.33%) patients’ C_0_/AUC_(0–12)_ ratios were below this threshold, and in 9 (10.70%) of these cases, IFTA was observed, while among the 60 patients with the C_0_/AUC_(0–12)_ ratio > 0.059, 25 (41.67%) corresponded to IFTA. This cut-off corresponds to a sensitivity of 73.53 % and a specificity of 70.00%.

**Figure 4:**
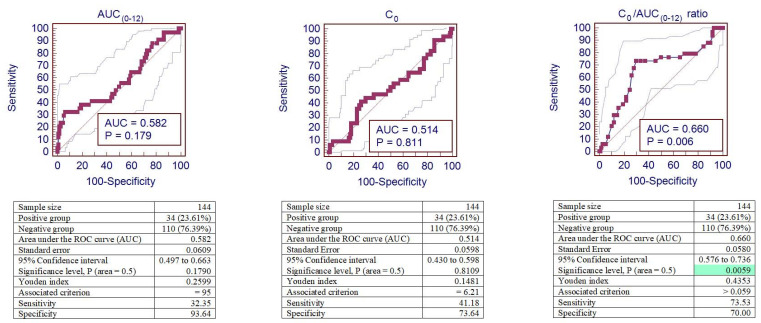
ROC curves of AUC_(0–12)_, C_0_, and the C_0_/AUC_(0–12)_ ratio for tacrolimus-induced IFTA. Analysis of sensitivity and specificity for IFTA represented by the ROC curves (area under the receiver operator curve—AUROC) in patients after kidney transplantation. The background green color remarks the significant area. IFTA—interstitial fibrosis and tubular atrophy.

Figure 4 shows the sensitivity and specificity analyses for AUC_(0–12)_, C_0_, and the C_0_/AUC_(0–12)_ ratio represented by the area under the receiver operator curve (AUROC) in kidney recipients with IFTA. Discrimination of the C_0_/AUC_(0–12)_ ratio showed reliable performance to predict IFTA after kidney transplantation, with an ROC curve of 0.660 (95% confidence interval (CI): 0.576–0.736), *p* < 0.01. However, ROC curves of AUC_(0–12)_ and C_0_ were not sufficiently informative for IFTA diagnosis *(p* > 0.05).

### 3.4. Comparison of C_0_ and AUC_(0–12)_ Methods for Assessing Cyclosporine Concentrations in Cyclosporine-Receiving Subjects with Post-Transplantation Time from 1 to 5 Years and with Post-Transplantation Time > 5 Years

#### 3.4.1. Analysis of the AUC_(0–12)_ Compliance within the Therapeutic Range

The evaluation of AUC_(0–12)_ demonstrated higher compliance within therapeutic range rates in the patients receiving only CsA (23.9–66.7%) versus the patients receiving CsA together with MMF, where compliance rates were 20.8–29.4%. Low dosing was more often observed in the patients receiving CsA together with MMF (35.4–51.0%), while higher rates of overdosing were observed in the patients receiving only CsA (47.8%) (Table 8).

Low CsA dosing, as well as low tacrolimus dosing is often associated with transplanted organ rejection. Patients whose AUC_(0–12)_ exposure values were below the range possibly had a higher transplanted organ rejection risk. This risk was increased in the patients who received either CsA monotherapy or CsA with MMF (35.4% vs. 51.0%). The results are presented in Table 8.

#### 3.4.2. Analysis of the C_0_ Compliance within the Therapeutic Range

Slightly different results were obtained while analyzing the C_0_ compliance within the therapeutic range. Compliance rates between the groups were quite similar and ranged from 63.0% to 71.9%. The most accurate dosing was observed in patients with grafts aged 1–5 years receiving CsA together with MMF (71.9%). Cases of CsA overdosing or cases where CsA C_0_ values were above the therapeutic range were observed in patients with grafts aged 1–5 years receiving CsA with MMF (19.6%) and in patients with grafts aged > 5 years, with an expected overdose of CsA of 5.2% (CsA + MMF) and 19.6% (CsA), respectively. The results are presented in Table 9.

The data showed that the AUC_(0–12)_ assessment was more related with CsA overdosing or low dosing than with accurate dosing, while the C_0_ assessment was related with accurate CsA dosing with compliance rates of 63.0–71.9%. The results are presented in Table 10 and Table 11.

#### 3.4.3. Independent Sample *t*-Test Analyses

**Figure 5:**
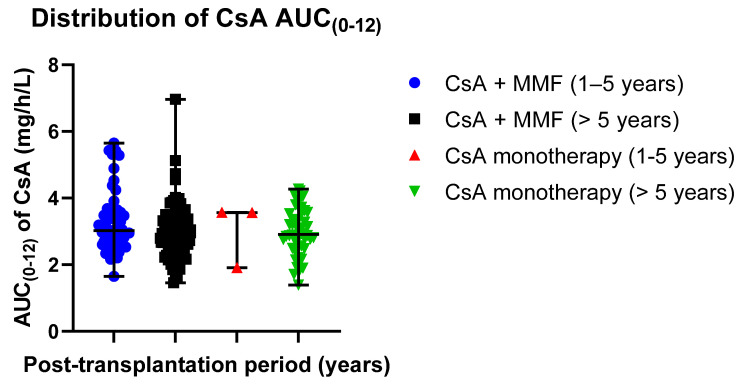
Distribution of the CsA AUC_(0–12)_ exposure values in the study groups: CsA + MMF and CsA monotherapy; MMF—mycophenolate mofetil; CsA—cyclosporine. Scatter plots display the individual AUC_(0–12)_ exposure values’ distribution from the median in CsA-receiving study groups (median ± SE): 3.02 ± 0.13 mg/h/L (CsA + MMF, post-transplantation time: 1–5 years) versus 3.56 ± 0.55 mg/h/L (CsA monotherapy, post-transplantation time: 1–5 years); 2.84 ± 0.80 mg/h/L (CsA + MMF, post-transplantation time > 5 years) versus 2.91 ± 0.10 mg/h/L (CsA monotherapy, post-transplantation time > 5 years). Respectively, the mean values (mean ± SD) were as follows: 3.31 ± 0.94 µg/h/L (Tacro + MMF, post-transplantation time: 1–5 years) versus 3.01 ± 0.96 µg/h/L (Tacro monotherapy, post-transplantation time: 1–5 years); 2.96 ± 0.79 µg/h/L (Tacro + MMF, post-transplantation time > 5 years) versus 2.98 ± 0.70 µg/h/L (Tacro monotherapy, post-transplantation time > 5 years).

The distribution of mean AUC_(0–12)_ exposure values in different groups was 3.31 ± 0.94 mg/h/L in patients receiving CsA with MMF (post-transplantation time: 1–5 years) and 2.96 ± 0.79 mg/h/L in patients receiving CsA monotherapy (post-transplantation time: 1–5 years); 3.01 ± 0.96 mg/h/L in patients receiving CsA with MMF (post-transplantation time > 5 years) and 2.98 ± 0.70 mg/h/L in patients receiving CsA monotherapy (post-transplantation time > 5 years) (Table 5). Data distribution is presented in scatter plots (Figure 5) displaying the individual AUC_(0–12)_ values’ distribution from the median in the CsA-receiving study groups (median ± SE): 3.02 ±0.13 mg/h/L (CsA + MMF, post-transplantation time: 1–5 years) versus 3.56 ± 0.55 mg/h/L (CsA monotherapy, post-transplantation time: 1–5 years); 2.84 ± 0.80 mg/h/L (CsA + MMF, post-transplantation time > 5 years) versus 2.91 ± 0.10 mg/h/L (CsA monotherapy, post-transplantation time > 5 years).

The independent sample *t*-test showed that the data statistically significantly differed between the groups receiving CsA with MMF. The patients having grafts aged 1–5 years received 10% higher CsA doses versus the patients with grafts aged > 5 years (204.41 ± 53.82 mg vs. 183.18 ± 50.14 mg) (*p* = 0.022; *p* < 0.05). Accordingly, the obtained mean C_0_ and AUC_(0–12)_ values statistically significantly differed. The mean AUC_(0–12)_ exposure value was 10% higher in the patients with grafts aged 1–5 years than in the patients with grafts aged > 5 years (3.31 ± 0.94 mg/h/L vs. 2.97 ± 0.79 mg/h/L) (*p* = 0.026; *p* < 0.05), while the mean C_0_ value was 23% higher in the patients with grafts aged 1–5 years versus the patients with grafts aged > 5 years (128.55 ± 51.84 µg/L vs. 97.90 ± 33.23 µg/L) (*p* < 0.05).

The independent sample *t*-test results also showed that the CsA dose statistically significantly differed between patients’ groups: patients receiving CsA + MMF (post-transplantation time > 5 years) had 10% higher CsA doses than patients receiving CsA monotherapy (post-transplantation time > 5 years) (183.18 ± 50.14 mg vs. 164.13 ± 48.55 mg) (*p* = 0.033; *p* < 0.05); patients receiving CsA monotherapy with post-transplantation time of 1–5 years had 30% higher CsA doses than patients receiving CsA monotherapy with post-transplantation time > 5 years (233.33 ± 28.87 mg vs. 164.13 ± 48.55 mg) (*p* = 0.036; *p* < 0.05) (Table 5). No other significant differences between these groups were noticed.

**Figure 6:**
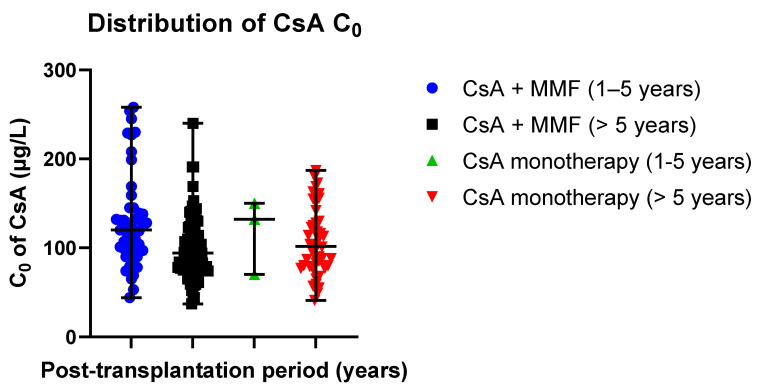
Distribution of CsA C_0_ values in the study groups: CsA + MMF and CsA monotherapy; MMF—mycophenolate mofetil; CsA—cyclosporine. Scatter plots display the individual C_0_ values’ distribution from the median in CsA-receiving study groups (median ± SE): 120.00 ± 7.26 µg/L (CsA + MMF, post-transplantation time: 1–5 years) versus 132.00 ± 24.23 µg/L (CsA monotherapy, post-transplantation time: 1–5 years); 94.00 ± 3.39 µg/L (CsA + MMF, post-transplantation time > 5 years) versus 101.50 ± 5.51 µg/L (CsA monotherapy, post-transplantation time > 5 years). Respectively, the mean values (mean ± SD) were as follows: 128.55 ± 51.84 µg/L (Tacro + MMF, post-transplantation time: 1–5 years) versus 117.33 ± 41.97 µg/L (Tacro monotherapy, post-transplantation time: 1–5 years); 97.90 ± 33.23 µg/L (Tacro + MMF, post-transplantation time > 5 years) versus 106.54 ± 37.39 µg h/L (Tacro monotherapy, post-transplantation time > 5 years).

Data distribution is presented in scatter plots (Figure 6) displaying the individual C_0_ values’ distribution from the median in CsA-receiving study groups (median ± SE): 120.00 ± 7.26 µg/L (CsA + MMF, post-transplantation time: 1–5 years) versus 132.00 ± 24.23 µg/L (CsA monotherapy, post-transplantation time: 1–5 years); 94.00 ± 3.39 µg/L (CsA + MMF, post-transplantation time > 5 years) versus 101.50 ± 5.51 µg/L (CsA monotherapy, post-transplantation time > 5 years).

#### 3.4.4. ANOVA Analysis

ANOVA analyses of four CsA receiving patients’ groups showed that only C_0_ and the CsA dose statistically significantly differed between the groups (*p* < 0.05) (Table 5).

### 3.5. Comparison of C_0_ and AUC_(0–12)_ Methods for Assessing Cyclosporine Concentrations in Cyclosporine-Receiving Patients with IFTA

#### 3.5.1. Rates of Non-Compliance

Non-compliance within the therapeutic range was associated with the appearance of IFTA. The results are presented in Table 10 and Table 11.

The assessment of AUC_(0–12)_ enabled to identify a larger number of subjects with IFTA than the assessment of C_0_. IFTA was obtained in 20 out of 96 patients receiving CsA + MMF (post-transplantation time > 5 years). Non-compliance was obtained in 8 out of 20 (40.0%) patients with AUC_(0–12)_ > 2.99 mg/h/L. At the same time, the assessment of C_0_ enabled to identify 2 out of 20 patients (10.0%) with C_0_ > 150 µg/h/L. Similar results were obtained in other CsA-receiving patients’ groups (Table 10 and Table 11).

#### 3.5.2. Cut-Offs for Non-Compliance

IFTA was observed in 39 (19.9%) patients out of 196 patients. ROC curve analyses were performed to identify the cut-off values of the increased risk of CsA-induced IFTA.

A cut-off of 2.34 mg/h/L was the optimal one to maximize AUC_(0–12)_ sensitivity and specificity for IFTA. According to this cut-off, 34 (17.35%) patients’ AUC_(0–12)_ values were below this threshold, and in 11 (32.40%) of these cases, IFTA was observed, while among the 162 patients with the AUC_(0–12)_ value > 2.34 mg/h/L, 28 (17.30%) corresponded to IFTA. This cut-off corresponds to a sensitivity of 28.21% and a specificity of 85.35%.

A cut-off of 120 µg/L was the optimal one to maximize C_0_ sensitivity and specificity for IFTA. According to this cut-off, 136 (69.39%) patients’ C_0_ values were below this threshold, and in 24 (17.60%) of these cases, IFTA was observed, while among the 60 patients with the C_0_ value > 120 µg/L, 15 (20.00%) corresponded to IFTA. This cut-off corresponds to a sensitivity of 38.46% and a specificity of 71.34%.

A cut-off of 36.755 was the optimal one to maximize the C_0_/AUC_(0–12)_ ratio’s sensitivity and specificity for IFTA. According to this cut-off, 122 (62.24%) patients’ C_0_/AUC_(0–12)_ ratios were below this threshold, and in 20 (16.40%) of these cases, IFTA was observed, while among the 74 patients with the C_0_/AUC_(0–12)_ ratio > 0.059, 19 (25.70%) corresponded to IFTA. This cut-off corresponds to a sensitivity of 48.72 % and a specificity of 64.97%.

**Figure 7:**
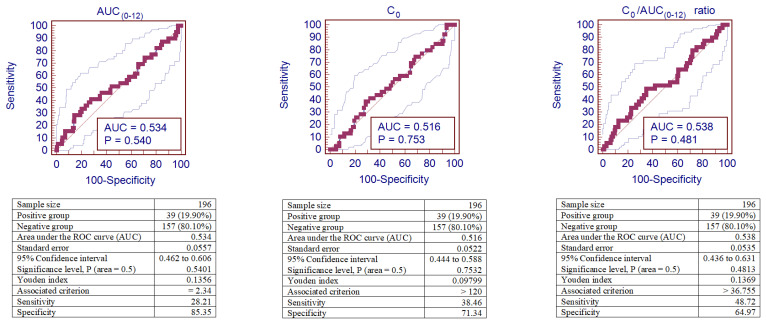
ROC curves of AUC_(0–12)_, C_0_, and the C_0_/AUC_(0–12)_ ratio for CsA-induced IFTA. Analysis of sensitivity and specificity for IFTA represented by the ROC curves (area under the receiver operator curve—AUROC) in patients after kidney transplantation. IFTA—interstitial fibrosis and tubular atrophy.

Figure 7 shows the sensitivity and specificity analyses for AUC_(0–12)_, C_0_, and the C_0_/AUC_(0–12)_ ratio represented by the area under the receiver operator curve (AUROC) in kidney recipients with IFTA. However, ROC curves of AUC_(0–12)_, C_0_, and the C_0_/AUC_(0–12)_ ratio were not sufficiently informative for IFTA diagnosis (*p* > 0.05).

## 4. Bland–Altman Plots for the Difference between C_0_ and AUC_(0–12)_

**Figure 8:**
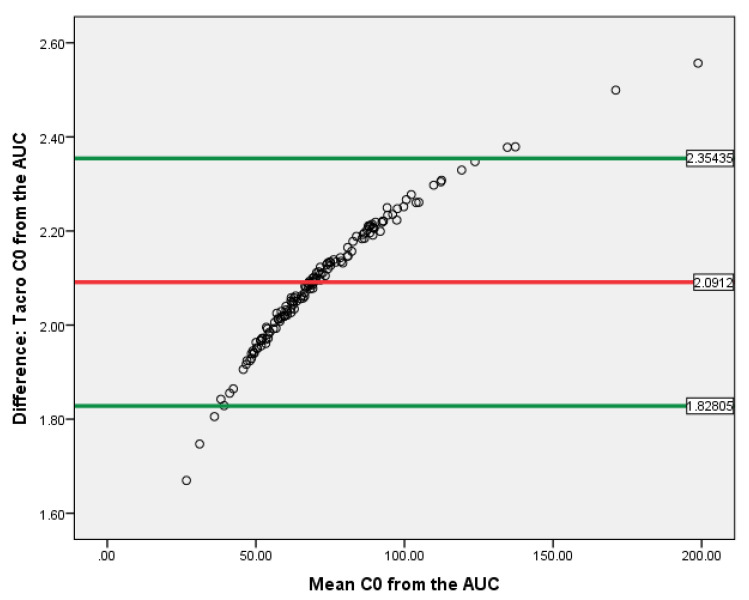
Bland–Altman plot for Tacro C_0_ and AUC_(0–12)_ in kidney recipients. The horizontal red line delineates the log-mean difference between C_0_ and AUC_(0–12)_, the upper and the lower horizontal green lines delineate ± 1.96 × standard deviation.

**Figure 9:**
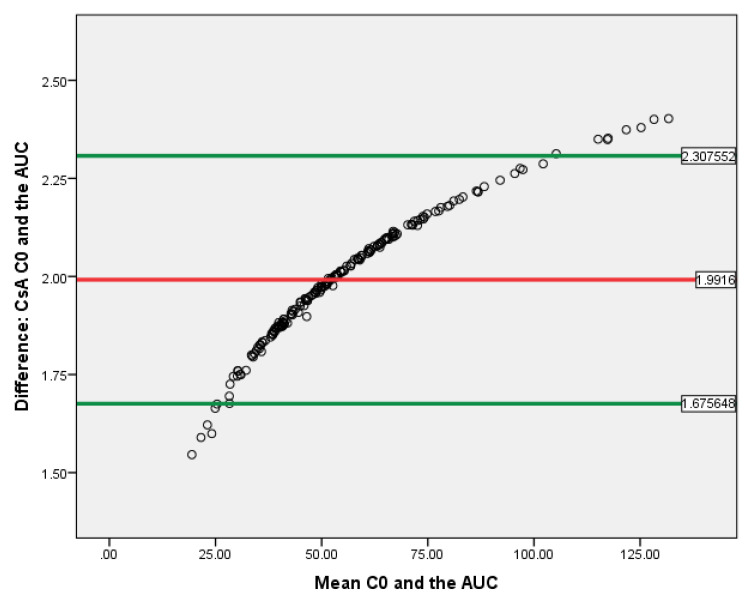
Bland–Altman plot for CsA C_0_ and AUC_(0–12)_ in kidney recipients. The horizontal red line delineates the log-mean difference between C_0_ and AUC_(0–12)_, the upper and the lower horizontal green lines delineate ± 1.96 × standard deviation.

T-test showed that the C_0_ and AUC_(0–12)_ difference correlated with either tacrolimus or cyclosporine exposure was evaluated. The data of Bland–Altman plot analyses are provided in Figure 8 and Figure 9. Both methods are proportionally biased (*p* < 0.01) and interchangeable.

## 5. Discussion

Many transplantation centers around the world use therapeutic drug monitoring (TDM) in clinical practice as a tool to achieve a predetermined target—drug concentration and to balance between efficacy and toxicity [18]. However, the relationship between the calcineurin inhibitor (CNI) dose exposure and nephrotoxicity is more complex than it used to be, and TDM no longer plays the important role in indicating the CNI-induced nephrotoxicity. On the other hand, the nonlinear relationship between doses and exposures leads to significant variability in the pharmacokinetics of CNI. A combination of demographic, clinical, genetic, and drug interaction data used to predict the algorithm would help to provide a more reliable dosage for the CNI [19]. Even if a predetermined therapeutic range is used, the occurrence of CNI-induced nephrotoxicity was limited, indicating the predictive value of TDM. Thus, the TDM performed in this study is undoubtedly significant in a broad sense.

It is important to assess the non-compliance within the therapeutic ranges of AUC_(0–12)_ of CsA and Tacro, and to correlate these deviations with the treatment outcomes. In the study conducted by K.A. Barraclough et al., the relationship between tacrolimus exposure AUC_(0–12)_ or C_0_ values and acute rejection was not observed. Although tacrolimus C_0_ values indicated in this study are sufficient and high, a large part of the subjects (38% on day 4 and 42% at 1 month) had the AUC_(0–12)_ exposure below the lower limit of the target range (150–250 µg/h/L). K.A. Barraclough et al. associated these results with a poor or average correlation between AUC_(0–12)_ and C_0_ or, consequently, with the inappropriate AUC_(0–12)_ target range [20]. The results obtained in this study were similar to the K.A. Barraclough et al. study results, despite the lower therapeutic range (120–150 µg/h) used in the K.A. Barraclough et al. study. The non-compliance rates were 44.4% in patients with post-transplantation time > 5 years receiving Tacro monotherapy; 60.0% in patients with post-transplantation time of 1–5 years receiving Tacro monotherapy; 45.5% in patients with post-transplantation time > 5 years receiving Tacro and MMF; and 31.1% in patients with post-transplantation time of 1–5 years receiving Tacro and MMF. The high non-compliance rates were possibly related to low dosing of tacrolimus.

The results of this study also showed that attention should be drawn to the fact that the assessment of AUC_(0–12)_ exposure values was made using the standard therapeutic range. The differentiation of the therapeutic range of AUC_(0–12)_ exposure values depending on the time after transplantation and the co-administered drug could possibly improve the results or lead to similar or better results than those obtained by assessing C_0_ compliance within therapeutic ranges.

The potential interaction between tacrolimus and MMF was considered based on the results of scarce in vitro or clinical studies [21,22,23]. The population pharmacokinetic drug interaction model demonstrated that the area under the concentration–time curve (AUC) of tacrolimus increased by 22.1% when it was co-administered with MMF in healthy volunteers [24]. Effect of interaction differed according to CYP3A5 genotypes [23]. CYP3A5 genotype was repeatedly identified as a significant covariate in previous population pharmacokinetic models of tacrolimus [24,25,26] Data of the published studies suggest that lower doses of tacrolimus are required to achieve adequate immunosuppression when used with MMF. However, the effect of interaction cannot be generalized to stable kidney transplant recipients from the data retrieved of the studies with healthy volunteers after a single dose of tacrolimus and MMF. More importantly, pharmacokinetic data from the tacrolimus administered as a single agent cannot be used to support the dose rationale if it is combined with MMF.

In MMF-treated organ transplant recipients, lower mycophenolic acid (MPA) plasma concentrations have been found in cyclosporine- compared with tacrolimus-based immunosuppressive regimens. CsA-mediated inhibition of the biliary excretion of MPAG (mycophenolic acid glucuronide) by the Mrp2 transporter is the mechanism responsible for the interaction between CsA and MMF [27].

New research also shows that proposed tacrolimus AUC target levels need to be redefined due to the circadian variation and flat real-life non-fasting PK-profiles. The association between high-peak concentrations and side effects of tacrolimus may be overestimated given the flat real-life non-fasting PK profiles. The effect of real-life dosing of tacrolimus may very well be present for other drugs and should be investigated for drugs where TDM is indicated [28]. K.A. Barraclough et al. also noted that the consensus guidelines, which refer to the target tacrolimus range of 150–250 µg/L [29] do not provide any guidance on the monitoring induction therapy or tacrolimus use together with other immunosuppressive agents [20].

The research shows that low tacrolimus dosing that does not correspond to the therapeutic range is associated with an increased risk of organ rejection [20] As the concentration of tacrolimus is closely related to graft survival [30,31], it is important to understand the relevant factors, including concomitant drug administration, which influence the variability of tacrolimus and to quantify their effects on the concentration of tacrolimus to assist in drug dosage decisions in patients [32].

M.A. Halim et al. concluded that the CsA dose should be individualized in renal transplant recipients, especially if they have viral hepatitis and a single daily dosing of cyclosporine has the advantage of decreasing dosage and CsA-related adverse effects while maintaining optimal graft function [4].

Thus, the research carried out and the results of this study show that TDM is undoubtedly significant in reducing the risk of organ rejection or overdose. Recent studies besides the AUC calculated by the Bayesian method offer tacrolimus sampling in the elimination phase (C_8_), contrary to C_0_ [33]; maintaining tacrolimus 2-h concentrations over 20 ng/mL for favorable long-term outcomes with minimal opportunistic infections [34]; assaying some Bayesian structure algorithms based on learning data-dependent scores (K2, TAN (Tree-Augmented Naive), Hill climber, Tabu); then, using the so called simple estimator in order to estimate the distribution of conditional probabilities while supporting a tool to find the best cyclosporine dose when switching between pharmaceutical forms [35]. The choice of methodology for TDM is certainly dependent on the capabilities of the research center, although the results of the studies show that the AUC_(0–12)_ calculated by the Bayesian method is a strong pharmacokinetic parameter [36] Furthermore, in the second consensus report for therapeutic drug monitoring of tacrolimus-personalized therapy, it is noted for the first time that the intra-patient variability must be evaluated; likewise, the monitoring of the C_0_/AUC ratio is proposed to identify those patients who are good candidates to analyze AUC-Tacro instead of C_0_ [37]. Therefore, the authors suggest evaluating this ratio at least once in the early period and once in the stable period for each transplant recipient [37].

## 6. Conclusions

Precise dosage was more often observed in patients with grafts aged 1–5 years receiving either cyclosporine or tacrolimus monotherapy than in patients with grafts aged > 5 years and receiving several medicaments. Therapeutic drug monitoring of tacrolimus performed by using AUC_(0–12)_ and C_0_ showed that tacrolimus is in most cases overdosed when considering C_0_, while the determination of AUC_(0–12)_ showed that tacrolimus is effectively dosed for 27.8–40.0% of the patients receiving only tacrolimus and for 25.0–31.9% of the patients receiving tacrolimus with MMF. In the 1–5 years post-transplantation group, a 10% higher CsA dose was observed, which was proportionate with a 10% higher AUC_(0–12)_ exposure value. This indicates good compatibility of the dosage and the AUC_(0–12)_ method.

The Bland–Altman plot demonstrated that C_0_ and AUC_(0–12)_ might be interchangeable methods, while the ROC curve analysis of the C_0_/AUC_(0–12)_ ratio in the tacrolimus-receiving patient group demonstrated reliable performance to predict IFTA after kidney transplantation, with the ROC curve of 0.660 (95% confidence interval (CI): 0.576–0.736), *p* < 0.01. Moreover, AUC_(0–12)_ and C_0_ of tacrolimus depend on the concomitant medication and adjustment of the therapeutic range for AUC_(0–12)_ might influence the results.

## Figures and Tables

**Table 1:** Therapeutic ranges of AUC_(0–12)_ and C_0_ for kidney recipients receiving tacrolimus or cyclosporine.

Groups	Therapeutic Ranges	
	AUC_(0–12)_	C_0_
1–5 years (Tacro + MMF)	120–150 µg/h/L	4–6 µg/L
>5 years (Tacro + MMF)	120–150 µg/h/L	3–5 µg/L
1–5 years (Tacro)	120–150 µg/h/L	4–6 µg/L
>5 years (Tacro)	120–150 µg/h/L	3–5 µg/L
1–5 years (CsA + MMF)	3.05–3.75 mg/h/L	75–150 µg/L
>5 years (CsA + MMF)	2.70–2.98 mg/h/L	75–150 µg/L
1–5 years (CsA)	3.05–3.75 mg/h/L	75–150 µg/L
>5 years (CsA)	2.70–2.98 mg/h/L	75–150 µg/L

CsA—cyclosporine; MMF—mycophenolate mofetil; Tacro—tacrolimus.

**Table 2:** Baseline data of tacrolimus-receiving patients.

Time after Transplantation	1–5 Years (Tacro + MMF)	>5 Years (Tacro + MMF)	1–5 Years (Tacro)	>5 Years (Tacro)	ANOVA
Age ± SD (years) (range)	51.70 ± 13.48 (19–75)	55.04 ± 10.57 (29–73)	52.42 ± 12.83 (34–75)	56.01 ± 10.89 (26–71)	*p* = 0.873
Tacro dose ± SD (mg) (range)	5.03 ± 2.47 (1.5–13)	4.58 ± 2.06 (2–9)	5.70 ± 2.83 (2–10)	5.44 ± 2.68 (1–10)	*p* = 0.430
Therapeutic range of the AUC_(0–12)_ exposure (µg/h/L)	120–150	120–150	120–150	120–150	-
AUC_(0–12)_ ± SD (µg/h/L) (range)	146.97 ± 53.04 (68–379)	129.73 ± 36.70 (59–235)	113.90 ± 17.95 (77–138)	129.72 ± 38.09 (50–195)	*p* = 0.055
Therapeutic range of the C_0_ concentration (µg/L)	4–6	3–5	4–6	3–5	-
C_0_ ± SD (µg/L) (range)	8.23 ± 2.81 (4.05–18.58)	7.23 ± 2.14 (3.13–13.63)	6.12 ± 1.47 (4.02–9.66)	7.54 ± 2.67 (3.26–13.81)	*p* = 0.022
Number of subjects	72	44	10	18	144

ANOVA—analysis of variance; CsA—cyclosporine; MMF—mycophenolate mofetil; Tacro—tacrolimus; SD—standard deviation.

**Table 3:** Comparative table of the Tacro AUC exposure values’ compliance within therapeutic ranges.

	Number of Subjects	1–5 Years (Tacro + MMF)	>5 Years (Tacro + MMF)	1–5 Years (Tacro)	>5 Years (Tacro)
		72	44	10	18
Therapeutic range	<120 µg/h/L	23 (31.9%)	20 (45.5%)	6 (60.0%)	8 (44.4%)
	120–150 µg/h/L	23 (31.9%)	11 (25.0%)	4 (40.0%)	5 (27.8%)
	>150 µg/h/L	26 (36.1%)	13 (29.5%)	-	5 (27.8%)

MMF—mycophenolate mofetil; Tacro—tacrolimus.

**Table 4:** Comparative table of Tacro C_0_ values’ compliance within therapeutic ranges.

	Number of Subjects	1–5 Years (Tacro + MMF)	>5 Years (Tacro + MMF)	1–5 Years (Tacro)	>5 Years (Tacro)
		72	44	10	18
Therapeutic range	<3 µg/L	NA	-	NA	-
	3–5 µg/L	NA	3 (6.8%)	NA	2 (11.1%)
	>5 µg/L	NA	41 (93.2%)	NA	16 (88.9%)
	<4 µg/L	-	NA	-	NA
	4–6 µg/L	13 (18.1%)	NA	6 (60.0%)	NA
	>6 µg/L	59 (81.9%)	NA	4 (40.0%)	NA

MMF—mycophenolate mofetil; Tacro—tacrolimus; NA—not applicable.

**Table 5:** Baseline data of cyclosporine-receiving patients.

Time after Transplantation	1–5 Years (CsA + MMF)	>5 Years (CsA + MMF)	1–5 Years (CsA)	>5 Years (CsA)	ANOVA
Age ± SD (years) (range)	55.01 ± 14.25 (22–78)	58.19 ± 13.53 (27–82)	58.97 ± 13.30 (44–69)	61.96 ± 13.59 (38–83)	*p* = 0.105
CsA dose ± SD (mg) (range)	204.41 ± 53.82 (120–400)	183.18 ± 50.14 (60–350)	233.33 ± 28.87 (200–250)	164.13 ± 48.55 (70–270)	*p* = 0.001
Therapeutic range of the AUC_(0–12)_ exposure (mg/h/L)	3.05–3.75	2.70–2.98	3.05–3.75	2.70–2.98	-
AUC_(0–12)_ ± SD (mg/h/L) (range)	3.31 ± 0.94 (1.65–5.65)	2.97 ± 0.79 (1.46–6.96)	3.01 ± 0.96 (1.91–3.57)	2.99 ± 0.70 (1.39–4.27)	*p* = 0.087
Therapeutic range of the C_0_ concentration (µg/L)	75–150	75–150	75–150	75–150	-
C_0_ ± SD (µg/L) (range)	128.55 ± 51.84 (44–258)	97.90 ± 33.23 (37–240)	117.33 ± 41.97 (70–150)	106.54 ± 37.40 (41–187)	*p* = 0.000
Number of subjects	51	96	3	46	196

ANOVA—analysis of variance; CsA—cyclosporine; MMF—mycophenolate mofetil; SD—standard deviation.

**Table 6:** Tacro AUC exposure values compliances within therapeutic ranges in patients with kidney alterations.

	Number of Subjects with IFTA	1–5 Years (Tacro + MMF)	>5 Years (Tacro + MMF)	1–5 Years (Tacro)	>5 Years (Tacro)	Total
		17 (23.6%) Out of 72	8 * (18.2%) Out of 44	3 (30.0%) Out of 10	6 (33.3%) Out of 18	34 (23.6%) Out of 144
Therapeutic range	<120 µg/h/L	5 (29.4%)	4 (50.0%)	2 (66.7%)	3 (50.0%)	14 (9.7%)
	120–150 µg/h/L	8 (47.1%)	1 (12.5%)	1 (33.3%)	1 (16.7%)	11 (7.6%)
	>150 µg/h/L	4 (23.5%)	3 (37.5%)	-	2 (33.3%)	9 (6.3%)

IFTA—interstitial fibrosis and tubular atrophy; MMF—mycophenolate mofetil; Tacro—tacrolimus; NA—not applicable. * 1 of the subjects experienced an adverse drug reaction.

**Table 7:** Tacro C_0_ compliances within therapeutic ranges in patients with kidney alterations.

	Number of Subjects with IFTA	1–5 Years (Tacro + MMF)	>5 Years (Tacro + MMF)	1–5 Years (Tacro)	>5 Years (Tacro)	Total
		17 (23.6%) Out of 72	8 * (18.2%) Out of 44	3 (30.0%) Out of 10	6 (33.3%) Out of 18	34 (23.6%) Out of 144
Therapeutic range	<3 µg/L	NA	-	NA	-	-
	3–5 µg/L	NA	1 (12.5%)	NA	1 (16.7%)	2 (5.9%)
	>5 µg/L	NA	7 (87.5%)	NA	5 (83.3%)	12 (35.3%)
	<4 µg/L	-	NA	-	NA	-
	4–6 µg/L	4 (23.5%)	NA	1 (33.3%)	NA	5 (14.7%)
	>6 µg/L	13 (76.5%)	NA	2 (66.7%)	NA	15 (44.1%)

IFTA—interstitial fibrosis and tubular atrophy; MMF—mycophenolate mofetil; Tacro—tacrolimus; NA—not applicable. * 1 of the subjects experienced an adverse drug reaction.

**Table 8:** Comparative table of CsA AUC exposure values compliances within therapeutic ranges.

	Number of Subjects	1–5 Years (CsA + MMF)	1–5 Years (CsA)	Number of Subjects	>5 Years (CsA + MMF)	>5 Years (CsA)
		51	3		96	46
Therapeutic range	<3.04 mg/h/L	26 (51.0%)	1 (33.3%)	<2.69 mg/h/L	34 (35.4%)	13 (28.3%)
	3.05–3.75 mg/h/L	15 (29.4%)	2 (66.7%)	2.70–2.98 mg/h/L	20 (20.8%)	11 (23.9%)
	>3.76 mg/h/L	10 (19.6%)	-	>2.99 mg/h/L	42 (43.8%)	22 (47.8%)

CsA—cyclosporine; MMF—mycophenolate mofetil.

**Table 9:** Comparative table of CsA C_0_ compliances within the therapeutic range.

	Number of Subjects	1–5 Years (CsA + MMF)	1–5 Years (CsA)	>5 Years (CsA + MMF)	>5 Years (CsA)
		51	3	96	46
Therapeutic range	<75 µg/L	6 (11.8%)	1 (33.3%)	22 (22.9%)	8 (17.4%)
	75–150 µg/L	35 (68.6%)	2 (66.7%)	69 (71.9%)	29 (63.0%)
	>150 µg/L	10 (19.6%)	-	5 (5.2%)	9 (19.6%)

CsA—cyclosporine; MMF—mycophenolate mofetil.

**Table 10:** CsA AUC _(0–12)_ compliances within therapeutic ranges in patients with IFTA (1–5 years and >5 years post-transplantation groups).

	Number of Subjects with IFTA	1–5 Years (CsA + MMF)	1–5 Years (CsA)	Number of Subjects with IFTA	>5 Years (CsA + MMF)	>5 Years (CsA)	Total
		10 (19.6%) Out of 51	1 (33.33%) Out of 3		20 (20.8%) Out of 96	8 (17.4%) Out of 46	39 (19.9%) Out of 196
Therapeutic ranges	< 3.04 mg/h/L	4 (40.0%)	-	< 2.69 mg/h/L	11 (55.0%)	2 (25.0%)	17 (8.7%)
	3.05–3.75 mg/h/L	3 (30.0%)	1 (100.0%)	2.70–2.98 mg/h/L	1 (5.0%)	3 (37.5%)	8 (4.1%)
	> 3.76 mg/h/L	3 (30.0%)	-	> 2.99 mg/h/L	8 (40.0%)	3 (37.5%)	14 (7.1%)

IFTA—interstitial fibrosis and tubular atrophy; CsA—cyclosporine; MMF—mycophenolate mofetil.

**Table 11:** CsA C_0_ compliances within the therapeutic range in patients with IFTA (1–5 years and >5 years post-transplantation groups).

	Number of Subjects with IFTA	1–5 Years (CsA + MMF)	1–5 Years (CsA)	>5 Years (CsA + MMF)	>5 Years (CsA)	Total
		10 (19.6%) Out of 51	1 (33.33%) Out of 3	20 (20.8%) Out of 96	8 (17.4%) Out of 46	39 (19.9%) Out of 196
Therapeutic range	< 75 µg/L	1 (10.0%)	-	5 (25.0%)	2 (25.0%)	8 (20.5%)
	75–150 µg/L	7 (70.0%)	1 (100.0%)	13 (65.0%)	5 (62.5%)	26 (66.7%)
	> 150 µg/L	2 (20.0%)	-	2 (10.0%)	1 (12.5%)	5 (12.8%)

IFTA—interstitial fibrosis and tubular atrophy; CsA—cyclosporine; MMF—mycophenolate mofetil.

## Abbreviations

AUC	area under the concentration time curve
BID	twice-daily dosing
C_0_	through level
CNI	calcineurin inhibitor
CsA	cyclosporine
IFTA	interstitial fibrosis and tubular atrophy
MMF	mycophenolate mofetil
PK	pharmacokinetic
Tacro	tacrolimus
TDM	therapeutic drug monitoring
SD	standard deviation
